# Endothelial Regulation by Exogenous Annexin A1 in Inflammatory Response and BBB Integrity Following Traumatic Brain Injury

**DOI:** 10.3389/fnins.2021.627110

**Published:** 2021-02-18

**Authors:** Han Liu, Junchi He, Yue Wu, Yang Du, Yinghua Jiang, Chengzhi Chen, Zhanyang Yu, Jianjun Zhong, Zhigang Wang, Chongjie Cheng, Xiaochuan Sun, Zhijian Huang

**Affiliations:** ^1^Department of Neurosurgery, The First Affiliated Hospital of Chongqing Medical University, Chongqing, China; ^2^Department of Neurosurgery, Qilu Hospital of Shandong University (Qingdao Campus), Qingdao, China; ^3^Department of Neurology, Xiangya Hospital, Central South University, Changsha, China; ^4^Departments of Radiology and Neurology, Massachusetts General Hospital, Harvard Medical School, Boston, MA, United States; ^5^Department of Occupational and Environmental Health, School of Public Health and Management, Research Center for Medicine and Social Development, Innovation Center for Social Risk Governance in Health, Chongqing Medical University, Chongqing, China

**Keywords:** annexin A1, blood–brain barrier, inflammation, RhoA, traumatic brain injury

## Abstract

**Background and Target:**

Following brain trauma, blood–brain barrier (BBB) disruption and inflammatory response are critical pathological steps contributing to secondary injury, leading to high mortality and morbidity. Both pathologies are closely associated with endothelial remodeling. In the present study, we concentrated on annexin A1 (ANXA1) as a novel regulator of endothelial function after traumatic brain injury.

**Methods:**

After establishing controlled cortical impact (CCI) model in male mice, human recombinant ANXA1 (rANXA1) was administered intravenously, followed by assessments of BBB integrity, brain edema, inflammatory response, and neurological deficits.

**Result:**

Animals treated with rANXA1 (1 μg/kg) at 1 h after CCI exhibited optimal BBB protection including alleviated BBB disruption and brain edema, as well as endothelial junction proteins loss. The infiltrated neutrophils and inflammatory cytokines were suppressed by rANXA1, consistent with decreased adhesive and transmigrating molecules from isolated microvessels. Moreover, rANXA1 attenuated the neurological deficits induced by CCI. We further found that the Ras homolog gene family member A (RhoA) inhibition has similar effect as rANXA1 in ameliorating brain injuries after CCI, whereas rANXA1 suppressed CCI-induced RhoA activation.

**Conclusion:**

Our findings suggest that the endothelial remodeling by exogenous rANXA1 corrects BBB disruption and inflammatory response through RhoA inhibition, hence improving functional outcomes in CCI mice.

## Background

Traumatic brain injury (TBI) has been a major cause of death and disability worldwide for decades (Wu et al., 2020). Although the mortality rate of patients with severe TBI has decreased by nearly 50% in the past 150 years, it is still as high as 30% (Stein et al., 2010). More than 30 TBI clinical trials have failed until now (Wright et al., 2014; Delbary-Gossart et al., 2016), and there is currently no effective treatment for TBI. Therefore, the research on TBI treatment strategy and prognosis has become the latest hotspot. Following initial impact, the secondary inflammatory response, blood–brain barrier (BBB) breakdown, and edema formation contribute to the clinical deterioration in patients, and the interplay between them has also been recognized. Robust inflammation can damage the endothelial junctions of normal BBB, and then BBB leakage permits movement of water from vasculature to the extracellular space in response to elevated osmotic gradient generated by the leakage of vascular components into the brain parenchyma, leading to vasogenic edema (Donkin and Vink, 2010). As a direct consequence of BBB leakage, brain edema is responsible for 50% of patients’ clinical deterioration following TBI (Feickert et al., 1999; Marmarou., 2003).

Annexin A1 (ANXA1) is dominantly expressed in the endothelium of the brain microvasculature and colocalizes with cellular cytoskeleton (Cristante et al., 2013). The BBB consists of tight and adherent junction complexes between endothelial cells. Thus, endothelial tight junction proteins (such as claudin-5, occludin, and ZO-1) and endothelial adherent proteins such as VE cadherin are essential to BBB integrity (Zhang et al., 2019). It was proven that ANXA1 is capable of regulating physiological and multiple sclerosis–conditioned BBB integrity and promoting cytoskeletal stability and enhancing endothelial junction protein expression by inhibiting the Ras homolog gene family member A (RhoA) (Cristante et al., 2013). RhoA is known to initiate the signaling pathway, resulting in the destabilization of the actin cytoskeleton, ultimately enhancing paracellular permeability (Terry et al., 2010). As a major downstream effector of RhoA, Rho-associated coiled-coil kinase (ROCK) regulates actin reorganization during cell adhesion, migration, contraction, and proliferation (Liu et al., 2018). On the other hand, ANXA1 acted to augment rolling velocity and reduce neutrophil adhesion to endothelium (Girol et al., 2013). ANXA1-null mice exhibited a higher extent of neutrophil extravasation, which is also associated with RhoA activity (Rao et al., 2017). Both pathological events are closely related to endothelial function; thus, we proposed a therapeutic potential of ANXA1 as an early regulator of vascular remodeling in TBI, putatively with the involvement of RhoA.

## Methods

### Animals and Controlled Cortical Impact Model

All animal procedures were conducted in compliance with the Animal Research: Reporting *In Vivo* Experiments (ARRIVE) guidelines–the National Institutes of Health Guide for the Care and Use of Laboratory Animals. All animal experiments were approved by the Animal Care and Use Committee of Chongqing Medical University.

Mice were 12 weeks old and weighed 20–22 g. The sham group received only craniotomy without cortical impact. Briefly, after deep isoflurane (3%) anesthesia, a midline longitudinal incision was performed; the skin was retracted to expose the skull. After establishing controlled cortical impact (CCI) model in male mice, a 5.0-mm-diameter craniotomy was established in the right parietal bone midway between bregma and lambda with the medial edge 1.0 mm lateral to the midline. Mice were impacted at 5.0 m/s with a 40-ms dwell time and 0.6-mm depression using a 3.0-mm-diameter convex tip, mimicking a moderate brain trauma in humans. Following surgery, bone wax was used to fill the hole on the skull, and the scalp was sutured closed. The animals were on an electric blanket to reduce mice suffering. They were held in an electric blanket to maintain body temperature until complete recovery from anesthesia. Then, human recombinant ANXA1 (rANXA1) (R&D, United States) was administrated through the tail vein after CCI. ROCK inhibitor Y27632 (20 nmol/kg, cytoskeleton) was injected into the contralateral ventricle (1 mm lateral and 0.5 mm backward to bregma on the skull surface) at 30 min pre-CCI (Huang et al., 2012).

### BBB Leakage

The integrity of BBB was reflected by Evans blue in the brain hemisphere following CCI. First, Evans blue dye (2% in saline, 4 mL/kg) was injected through tail vein 1 h before being sacrificed. Under deep anesthesia, the chest wall was opened, and mice were perfused with phosphate-buffered saline (PBS) intracardially. After decapitation, brain hemispheres were removed, weighed, and homogenized in trichloroacetic acid and centrifuged at 14,000 rpm for 15 min. Then, the resultant supernatants were separated and analyzed at 630 nm for excitation and 680 nm for emission using a spectrophotometer (SpectraMax M5). The Evans blue amount was quantified according to a linear standard curve.

The quantification of extravasated immunoglobulin G (IgG) molecules was determined by immunohistochemical staining, modified from the previous way (Zhang et al., 2016; Thomas et al., 2020). Brain slices 500 μm apart (total nine sections in each animal to cover lesion cavity) were blocked with 5% donkey serum in PBS with 0.05% Tween 20 for 1 h. Brain slices were incubated with Biotin-SP AffiniPure F(ab’)2 Fragment Donkey Anti-Mouse IgG (H + L) antibody (1:200; Jackson Immunoresearch Laboratories, United States) at 4°C overnight, followed by reaction with fluorescein streptavidin (1:200, Jackson Immunoresearch Laboratories, United States). The IgG immunointensity in injured hemisphere of each level was calculated by ImageJ software and added as final value, while contralateral hemisphere as negative control. The IgG-positive area was observed under fluorescence microscope.

### Water Content

These specimens were dried in an oven at 100°C for 48 h. The following formula was used to calculate the percentage of water content in hemispheres: ([wet weight−dry weight]/wet weight) × 100%.

### Immunohistochemistry

First, coronal brain slices above were fixed with 4% paraformaldehyde and then blocked with 5% donkey serum for 60 min. After incubation overnight at 4°C with rat antineutrophil (1:200, Abcam), slides were scanned with fluorescence microscope (ECLIPSE Ti-s, Nikon, Japan). For quantification, the number of neutrophil cells per 20× field in the perilesion area was counted in four randomized fields (two in cortex and two in subcortex) of each animal. And the immunoreactivity of IgG area per 20× field in the perilesion area was calculated in four randomized fields (two in cortex and two in subcortex) in each animal.

### Immunoblots

Brain tissue dissected from injured cortex within the injured hemisphere or from the left hemisphere of uninjured control mice was homogenized on ice in RIPA (radioimmunoprecipitation assay) buffer (Beyotime, China) containing protease inhibitor cocktail (Sigma–Aldrich). Homogenates were centrifuged at 14,000 revolutions/min (rpm) for 15 min at 4°C. Protein content of the supernatant was assayed (Bio-Rad Laboratories, United States), and aliquots of protein were boiled in denaturing sample buffer (62.5 mmol/L Tris pH 6.8, 2% sodium dodecyl sulfate, 5 mmol/L ethylenediaminetetraacetic acid, 10% glycerol, 0.25% 2-mercaptoethanol, 0.01% bromophenol blue). The protein samples were denatured by boiling and were resolved (20 μg) on 6 or 10% sodium dodecyl sulfate–polyacrylamide gels (Invitrogen) and blotted onto polyvinylidene difluoride membranes (Millipore). GAPDH was used as an internal reference. Membranes were blocked for 1 h in 5% milk in Tris-buffered saline (pH 7.4) containing 0.1% Tween 20 and then incubated overnight at 4°C with primary antibodies: GAPDH (Cell Signaling, 1:1,000), ANXA1 (Abcam, 1:500), ZO-1 (Santa Cruz, 1:1,000), occludin (Santa Cruz, 1:1,000), VE cadherin (Santa Cruz, 1:1,000), claudin-5 (Abcam, 1:1,000), RhoA (Santa Cruz, 1:2,000), vascular cell adhesion molecule 1 (VCAM-1) (Santa Cruz, 1:1,000), intercellular adhesion molecule 1 (ICAM-1) (Santa Cruz, 1:1,000), E-selectin (Santa Cruz, 1:1,000), and CD99 (Abcam, 1:1,000). Membranes were washed in Tris-buffered saline Tween 20 and then incubated for 1 h with an appropriate horseradish peroxidase–conjugated secondary antibody (1:20,000 in Tris-buffered saline Tween 20, 5% milk) at room temperature. Proteins of interest were detected using the enhanced chemiluminescence kit (Beyotime, China) and filmed. The optical densities for protein bands were analyzed quantified with Quantity One 4.6.2 (Bio-Rad Laboratories, United States). Three independent experiments were carried out to verify protein expression.

### RhoA Activation Assay

GTP-RhoA and total-RhoA were extracted from injured cortex tissue according to the instruction of Rho Activation Assay Kits (Cytoskeleton, United States). For total RhoA expression, 40 μg of total protein was used. Activated/total RhoA was detected using an anti-RhoA antibody (Cytoskeleton). RhoA activity semiquantification was determined by using ImageJ and expressed as the ratio between RhoA/total RhoA. Results were expressed as a relative ratio, normalized to the mean value of the sham group.

### Enzyme-Linked Immunosorbent Assay

After harvest of injured cortex tissue, the homogenate was centrifuged at 5,000 × *g* for 5 min. The supernatant was collected and prepared for subsequent assay. Interleukin-1β (IL-1β) and tumor necrosis factor α (TNF-α) concentrations were measured using commercially available enzyme-linked immunosorbent assay (ELISA) kits (USCN Life Science, China) according to the protocols of the kits. Briefly, the samples were added to each well of monoclonal anti-mouse IL-1β or TNF-α antibody-coated microtiter plates (ELISA plates) for 30 min at 37°C. Unbound material was washed off, and bound antibody was detected by addition of horseradish peroxidase for 15 min at 37°C. Absorbance was measured 15 min after addition of substrate. A standard curve was constructed using various dilutions of IL-1β and TNF-α.

### Microvessel Isolation

Microvessel isolation was conducted as previously described (Corvol et al., 2018). The mice were sacrificed 24 h after injury. After perfusion with PBS, the brains were harvested. Two sides of hemispheres (contralateral side and ipsilateral side) were separated; the core lesion areas (which fall apart quite easily) and undermined white matter were removed, rolling on the filter paper to get rid of meninges and the remaining loose debris. After this, cortical tissue from each hemisphere was pooled, homogenized in cold PBS on ice, and then centrifuged at 4°C, 1,000 × *g* for 5 min. The tissue pellet was suspended in PBS and then centrifuged again at 4°C, 1,500 × *g* for 20 min. The resuspended pellet with PBS was filtered with cell strainer (70 μm) to get rid of the debris. The extracted tissue from six mice merged into each sample.

### Behavioral Tests

Before and after the CCI model (days −1, 1, 3, 7, 14, and 28), the neurological functions of the mice were evaluated using a set of neurobehavioral tasks [neurological severity score (NSS)]. Briefly, NSS test was carried out as Tsenter’s protocol (Tsenter et al., 2008). A ten-point NSS was applied. The neuroscore consisted of 10 different tasks evaluating motor ability, alertness, balancing, and general behavior. One point was awarded for failure to complete a task.

Furthermore, the Morris water maze was applied to evaluate cognitive function from 15 to 21 days post-CCI as previously described (Huang et al., 2016). Briefly, five consecutive daily training sessions were performed for learning latency. For each trial, mice were randomized to start from one of four directions (north, south, east, or west) to find the submerged platform within 90 s. Mice that failed to reach the platform within 90 s were placed on the platform by the researcher and allowed to remain there for 15 s. In the probe trial, staying period and entry times of the platform area and target quadrant were recorded at day 21. To exclude the potential difference of visual ability between groups, extra visible trial was performed using a labeled platform above the water level. The behavior assessments as mentioned previously were performed by an independent investigator who was blind to the group assignment.

### Lesion Volume

At 28 days after CCI, the animals were perfused with 0.1 mol/L PBS under deep anesthesia. Brains were frozen-sectioned at the thickness of 10 μm. Brain slices 500 μm apart were stained with hematoxylin-eosin then photographed. The target level was between 1 mm anterior and 3 mm posterior to the bregma. Brain slices 500 μm apart (total nine sections in each animal) were collected to cover the core lesion area. The volume of injured tissue was measured with ImageJ 1.48 software. Damaged tissue volume = contralateral hemisphere volume−ipsilateral hemisphere volume.

### Statistical Analysis

Data are presented as mean ± SD. The generated data were normally distributed (Zhao et al., 2017). All the analyses were performed with the SigmaStat software (San Jose, CA, United States). Immunoblot and immunohistochemistry data were analyzed by one-way analysis of variance (ANOVA) followed by Tukey–Kramer *post hoc* tests. Neurobehavioral assessments were analyzed by repeated-measures ANOVA (Tornero et al., 2013; Zemmar et al., 2014; Delbary-Gossart et al., 2016), followed by Tukey *post hoc* tests. One-way ANOVA followed by *post hoc* test was used to evaluate the statistical differences in each time point between groups. The most conservative multiple-test correction was applied using the Bonferroni method. For all analyses, *p* < 0.05 was considered significant.

## Results

### ANXA1 Expression Increased in the Perilesion Cortex After TBI

**FIGURE 1:**
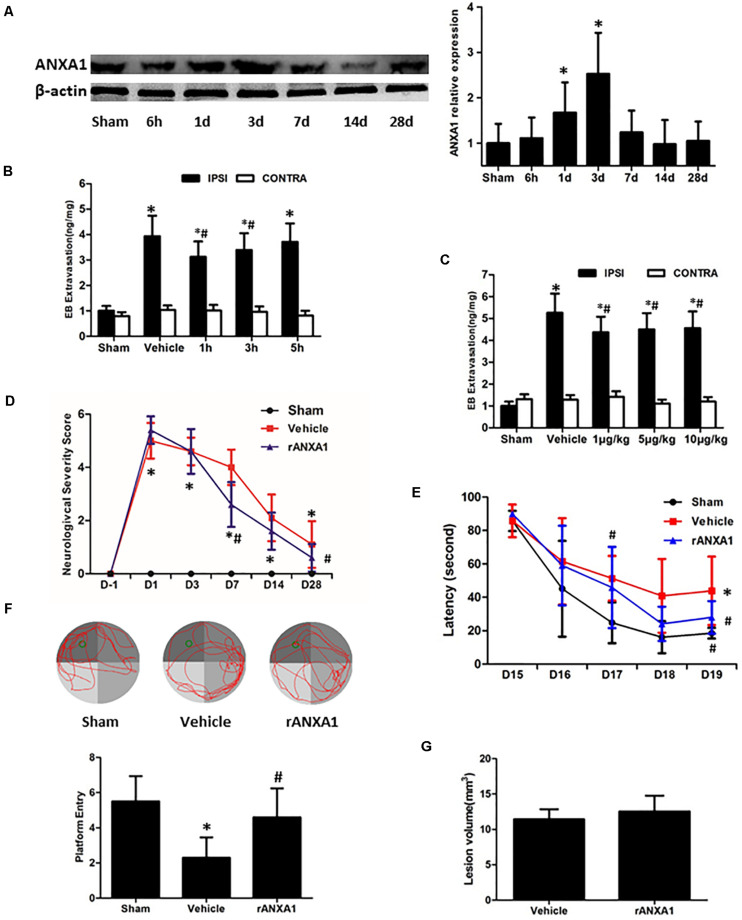
Effects of rANXA1 administration on BBB integrity after CCI. **(A)** Temporal expression of ANXA1 after CCI. ANXA1 expression increased at day 1 after CCI (*p* < 0.05) and the elevation peaked at day 3 (*p* < 0.05). After that, the expression declined and returned to baseline at day 14. Representative bands and quantitative analysis of endogenous ANXA1 expression in the ipsilateral cortex. Relative density of each protein has been normalized against the sham group. *p < 0.05 vs. sham, *n* = 5 per group. **(B,C)** Effects of rANXA1 administration on BBB integrity after CCI. rANXA1 treatment 1 h post-CCI protocol induced more decrease in Evans blue amount than 3 h post-CCI. Therefore, 1 h post-CCI was chosen as the optimal time point for drug administration in the following study. Vehicle group was treated 1 h post-CCI **(B)**. Serial doses of rANXA1 ranging from 1 to 10 μg/kg were efficient to prevent Evans blue leakage (*p* < 0.05). *p < 0.05 vs. sham, #*p* < 0.05 vs. vehicle, *n* = 8 per group. Effects of rANXA1 on functional outcomes and lesion size after CCI **(D–G)**. The animals treated with rANXA1 exhibited significant overall improvement in these examinations compared with vehicle group (*p* < 0.05). Compared with the vehicle-treated mice, rANXA1 failed to change the lesion volume significantly after CCI (*p* > 0.05). Neurological severity scores before and after CCI **(D)**. Learning latency **(E)** and probe trial **(F)** in Morris water maze after CCI. **(G)** Lesion volume at 28 days after CCI. *p < 0.05 vs. sham, #*p* < 0.05 vs. vehicle, *n* = 10 per group.

To verify the endogenous reaction of ANXA1 to brain trauma, we measured its expression in the injured hemisphere of CCI mice at different time points (Figure 1A). The immunoblots showed a delayed increase of the ANXA1 level after injury. Compared with the sham-operated animals, there was a 1.7-fold increase of ANXA1 expression at day 1 after CCI (*p* < 0.05), and the elevation peaked at day 3 (*p* < 0.05). After that, the expression declined and returned to baseline at day 14.

### Effects of rANXA1 Administration on BBB Disruption, Neurological Deficits, and Lesion Size After CCI

To assess the precise and optimal effects of ANXA1 on BBB permeability after CCI, we examined Evans blue extravasation in bilateral hemispheres at 24 h after CCI with different timing (1, 3, and 5 h post-surgery, Figure 1B) and graded dose (1, 5, and 10 μg/kg) of rANXA1 administration (Figure 1C). After CCI, the leakage of Evans blue was detected in the ipsilateral hemispheres. Our results showed that rANXA1 treatment (1 μg/kg) at 1, 3, and 5 h post-CCI significantly ameliorated dye extravasation compared to the vehicle group (*p* < 0.05) and 1 h post-CCI protocol induced the least decrease in Evans blue amount. We thus chose 1 h post-CCI as the optimal time point for drug administration. We applied serial doses of rANXA1 ranging from 1 to 10 μg/kg, and the results show that each dose was efficient to prevent Evans blue leakage (*p* < 0.05). Because the initial dose (1 μg/kg) seemed to exert significant effect in BBB protection, and further increase in concentration failed to enhance its efficiency, this administrative setting (1 μg/kg at 1 h post-CCI) was selected for subsequent experiments.

To determine the gross effects of human rANXA1 on the functional recovery after CCI, we compared the performances of animals in NSS and Morris water maze tests (Figures 1D–F). After CCI, the neurological impairments were observed in all the tests. Consistently, the animals treated with rANXA1 exhibited significant overall improvement in these examinations compared with vehicle group (*p* < 0.05). To examine if rANXA1 contributes to the brain tissue damage after CCI, the volume of lesion cavity was measured at 28 days after CCI (Figure 1G). Compared with the vehicle-treated mice, rANXA1 failed to significantly change the lesion volume after CCI (*p* > 0.05).

### Effects of rANXA1 Administration on BBB Permeability and Brain Edema After CCI

**FIGURE 2:**
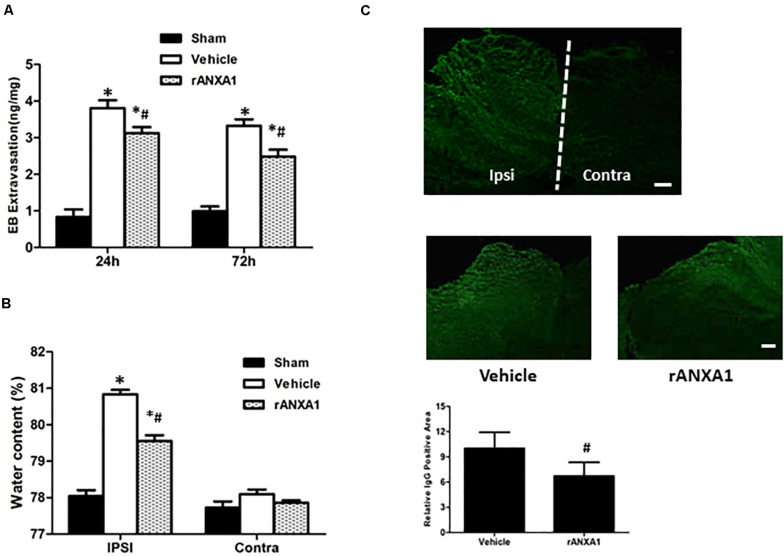
Effects of rANXA1 on BBB disruption after CCI. **(A)** Quantitation of extravasated Evans blue at 24 and 72 h after CCI and **(B)** quantitation of hemispherical water content at 24 h after CCI. After rANXA1 treatment, less extravasated dye was detected in injured hemisphere until 72 h following CCI (*p* < 0.05). Following CCI, the water content in the vehicle group increased remarkably in ipsilateral hemisphere. This disruption was attenuated by rANXA1 treatment with significance (*p* < 0.05). *p < 0.05 vs. sham, #*p* < 0.05 vs. vehicle, *n* = 8 per group. **(C)** Effects of rANXA1 on BBB disruption after CCI. Representative images and quantitative analysis of IgG fluorescence surrounding the lesion area at 24 h after CCI. Under fluorescence microscope, plasma-derived IgG leakage was observed in the ipsilateral cortex, striatum, and hippocampus, whereas the contralateral hemisphere was comparatively normal. rANXA1 treatment significantly reduced IgG extravasation area as compared to vehicle. Relative immunoactivity was normalized to the sham group. *p < 0.05 vs. sham, #*p* < 0.05 vs. vehicle, *n* = 5 per group. Scale bar = 100 μm.

To learn the dynamic and persistent bioactivity from rANXA1 in BBB protection, similar experiments were repeated at 24 and 72 h after CCI separately (Figure 2A). After rANXA1 treatment, less extravasated dye was detected in injured hemisphere until 72 h following CCI (*p* < 0.05). Given that BBB damage may lead to vasogenic cerebral edema, we next assessed the ability of rANXA1 at post-traumatic brain water content (Figure 2B). Following CCI, the water content in the vehicle group increased remarkably in the ipsilateral hemisphere. This disruption was attenuated by rANXA1 treatment with significance (*p* < 0.05).

The spatial profile of BBB protection by rANXA1 was additionally measured at 24 h after CCI. Under fluorescence microscope, plasma-derived IgG leakage was observed in the ipsilateral cortex, striatum, and hippocampus following CCI, whereas the contralateral hemisphere was comparatively normal (Figure 2C). And rANXA1 treatment significantly reduced IgG extravasation area as compared to vehicle (Figure 2C).

### Effects of rANXA1 Administration on Junctional Proteins After CCI

**FIGURE 3:**
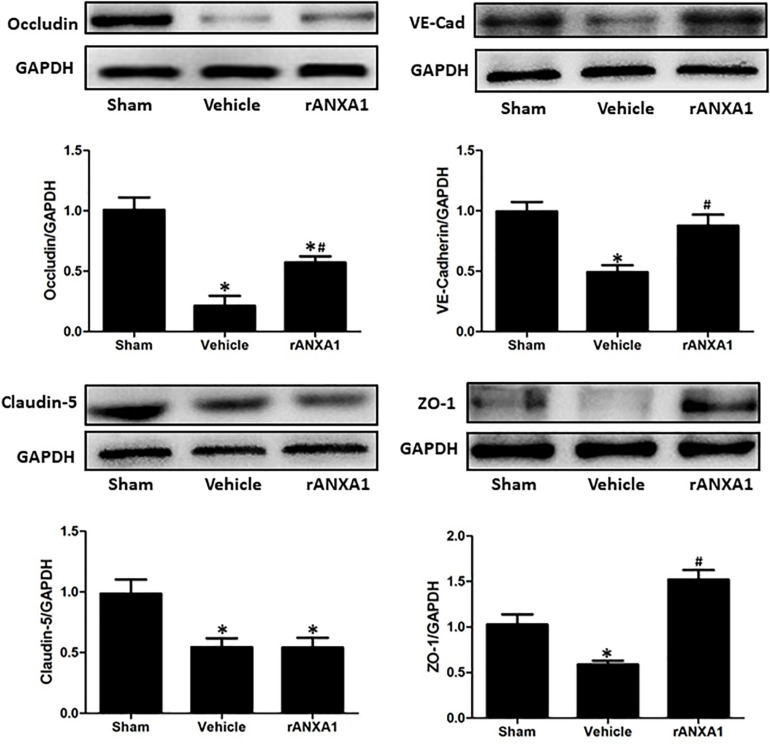
Effects of rANXA1 on the expressions of endothelial junctional proteins after CCI. The expressions of junctional proteins (occludin, claudin-5, ZO-1, and VE-cadherin) were significantly reduced at 24 h post-CCI (*p* < 0.05). However, rANXA1 preserved the expression levels of occludin, ZO-1, and VE-cadherin (*p* < 0.05), rather than claudin-5 (*p* > 0.05). Representative bands and quantitative analysis of the expressions of junctional proteins at 24 h after CCI. Relative density of each protein was normalized to the sham group. *p < 0.05 vs. sham, #*p* < 0.05 vs. vehicle, *n* = 5 per group.

To elucidate the structural alteration of BBB following CCI, the integrity of tight and adherent junction was further assessed. The expressions of junctional proteins (Occludin, claudin-5, ZO-1, and VE cadherin) were significantly reduced at 24 h post-CCI (*p* < 0.05) (Figure 3). However, rANXA1 preserved the expression levels of occludin, ZO-1, and VE-cadherin (*p* < 0.05), rather than claudin-5 (*p* > 0.05).

### Effects of rANXA1 Administration on Inflammatory Response and Endothelial Molecules After CCI

**FIGURE 4:**
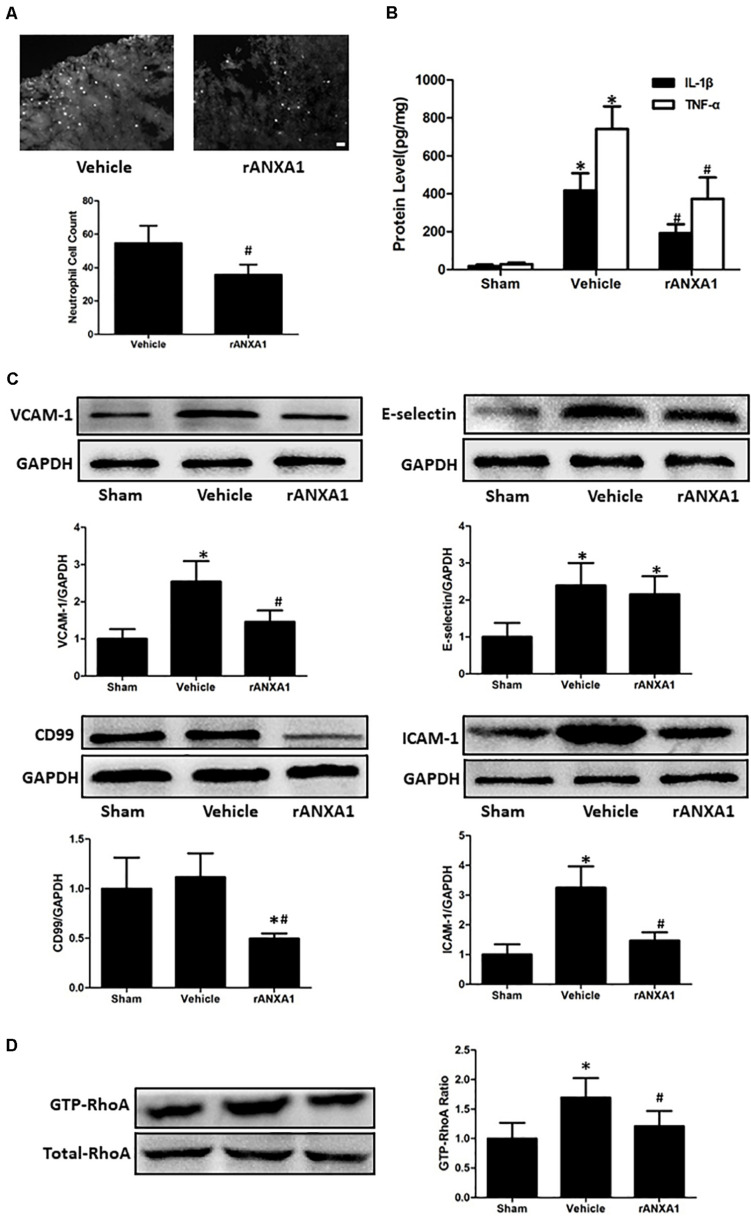
Effects of rANXA1 on systemic infiltrating inflammation after CCI **(A–C)**. **(A)** Representative images and quantitative analysis of neutrophil infiltration surrounding the lesion area at 72 h after CCI. Brain neutrophil number surrounding the lesion area significantly decreased after rANXA1 treatment (*p* < 0.05). **(B)** Quantitative analysis of the protein levels of inflammatory cytokines at 72 h after CCI. rANXA1 significantly reduced the expression of IL-1β and TNF-α compared to the vehicle group (*p* < 0.05). **(C)**. Representative bands and quantitative analysis of the expressions of endothelial molecules at 72 h after CCI. CCI resulted in a remarkable increase in VCAM-1, ICAM-1, and E-selectin expressions (*p* < 0.05). The protein levels of VCAM-1 and ICAM-1 were significantly declined after rANXA1 treatment (*p* < 0.05). It did not induce any statistical difference in E-selectin expression (*p* > 0.05). Moreover, rANXA1 significantly suppressed the expression of CD99 within endothelial cells (*p* < 0.05). **(D)** Effects of rANXA1 on RhoA activity after CCI. The expression of activated RhoA (GTP-RhoA) was enhanced at 24 h post-CCI. And the rAXNA1 treatment significantly inhibited RhoA activation as compared to vehicle (*p* < 0.05). *p < 0.05 vs. sham, #*p* < 0.05 vs. vehicle, *n* = 8 per group in panel **(A)**, scale bar = 100 μm; *n* = 6 per group in panel **(B)**; *n* = 5 per group in panels **(C,D)**.

To assess the potential effects of rANXA1 on inflammatory response, we measured neutrophil counts in injured cortex at 72 h in mice pretreated with rANXA1 or vehicle (Figure 4A). It showed that brain neutrophil number surrounding the lesion area significantly decreased after rANXA1 treatment (*p* < 0.05). In addition, we actually measured the temporal changes of neutrophil infiltration on the preinjury day and days 1, 3, and 7 post-CCI (Supplementary Figure 1). We almost did not see any infiltrated neutrophil in the sham group. And the results also showed that brain neutrophil number surrounding the lesion area increased maximally on day 3 (*p* < 0.05). Moreover, we examined the protein levels of classical proinflammatory cytokines at 72 h post-CCI by ELISA (Figure 4B). It showed that rANXA1 significantly reduced the expression of IL-1β and TNF-α compared to the vehicle group (*p* < 0.05).

To explore the possible mechanism involved in ANXA1-associated anti-inflammation effect, the microvessels were isolated to analyze the expression of endothelial molecules at 72 h post-CCI (Figure 4C). CCI resulted in a remarkable increase in VCAM-1, ICAM-1, and E-selectin expressions (*p* < 0.05). The protein levels of VCAM-1 and ICAM-1 were significantly declined after rANXA1 treatment (*p* < 0.05). It did not induce any statistical difference in E-selectin expression (*p* > 0.05). Moreover, rANXA1 significantly suppressed the expression of CD99 within endothelial cells (*p* < 0.05).

### ANXA1 Treatment Inhibited RhoA Activity and Thereby Modulated Endothelial Function

To verify the signaling pathway potentiating ANXA1-driven BBB protection, the RhoA activation (GTP-RhoA/RhoA ratio) was investigated before and after CCI (Figure 4D). The expression of activated RhoA (GTP-RhoA) was enhanced at 24 h post-CCI. And the rAXNA1 treatment significantly inhibited RhoA activation as compared to vehicle (*p* < 0.05).

**FIGURE 5:**
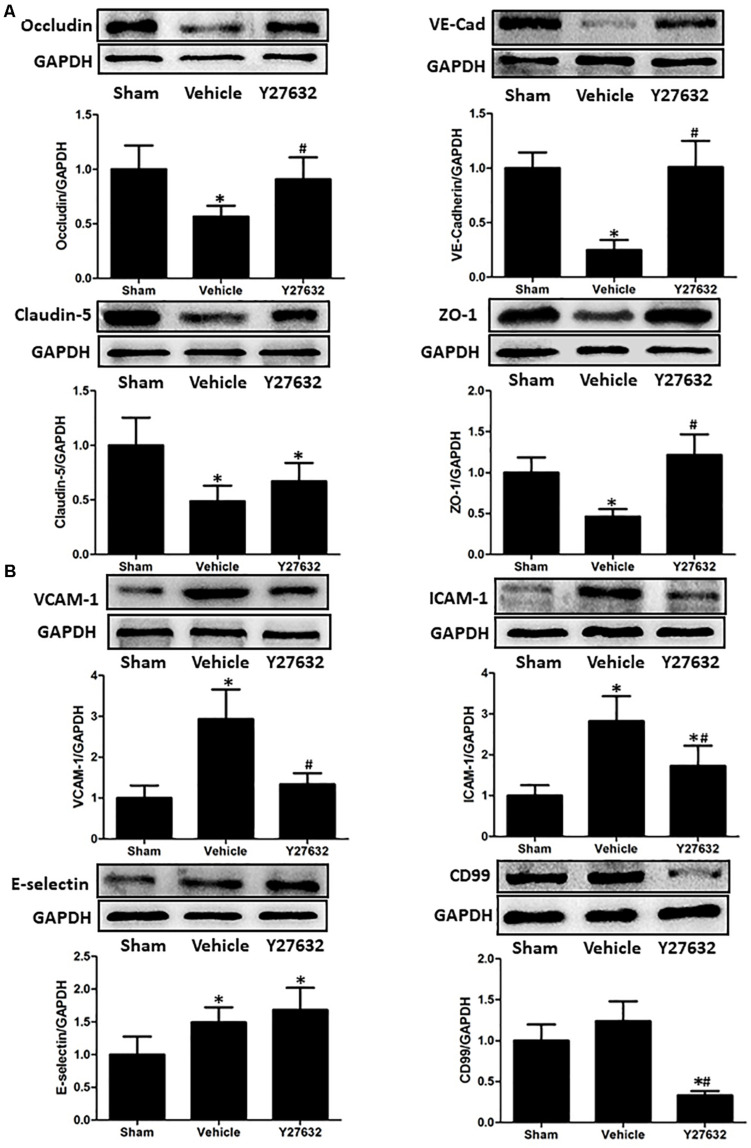
Effects of Y27632 on endothelial function after CCI. **(A)** Representative bands and quantitative analysis of the expressions of occludin, VE-cadherin, claudin-5, and ZO-1 at 24 h after CCI. Y27632 preserved the expression levels of occludin, ZO-1, and VE-cadherin (*p* < 0.05), rather than claudin-5 (*p* > 0.05). **(B)** Representative bands and quantitative analysis of the expressions of endothelial molecules at 72 h after CCI. The endothelial molecules from isolated microvessels were analyzed. The protein levels of VCAM-1, ICAM-1, and CD99 were significantly suppressed by Y27632 treatment (*p* < 0.05). Relative density of each protein was normalized to the sham group. *p < 0.05 vs. sham, #*p* < 0.05 vs. vehicle, *n* = 5 per group.

The bioactivities of RhoA on endothelial function were evaluated by ROCK inhibitor Y27632 (Figures 5A,B). First, the expressions of junctional proteins were repeatedly measured, and Y27632 preserved the expression levels of occludin, ZO-1, and VE-cadherin (*p* < 0.05), rather than claudin-5 (*p* > 0.05). Then, endothelial molecules from isolated microvessels were analyzed by immunoblot. The protein levels of VCAM-1, ICAM-1, and CD99 were significantly suppressed by Y27632 treatment (*p* < 0.05).

### RhoA Inhibition Exhibited Similar Effects in Ameliorating Brain Injuries After CCI as rANXA1 Administration

**FIGURE 6:**
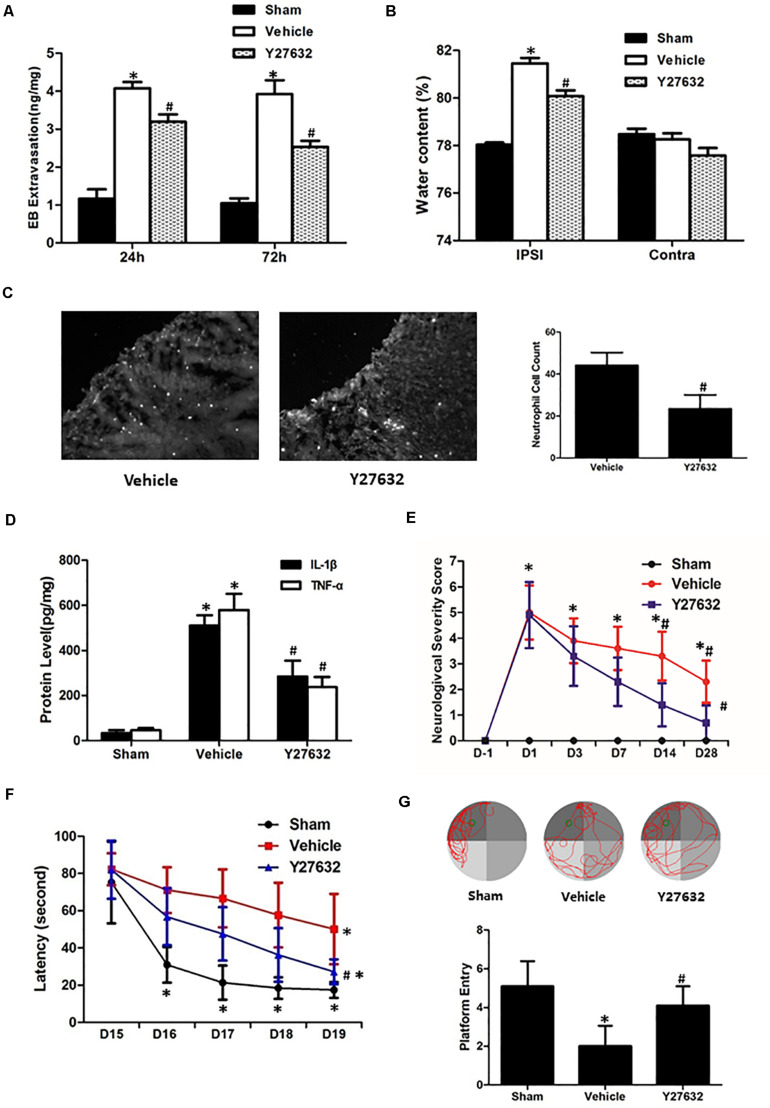
Effects of Y27632 on secondary brain injuries after CCI. **(A)** Y27632 induced less extravasated dye in injured hemisphere at both 24 and 72 h post-CCI (*p* < 0.05). **(B)** Y27632 attenuated post-traumatic brain water content with significance (*p* < 0.05). **(C)** Neutrophil number surrounding the lesion area was significantly decreased by Y27632 treatment (*p* < 0.05). **(D)** Y27632 significantly reduced protein levels of IL-1β and TNF-α as compared to the vehicle group (*p* < 0.05). **(E)** Neurological severity scores before and after CCI, **(F)** learning latency, and **(G)** probe trial in Morris water maze after CCI. The functional results showed Y27632 induced the overall improvements in all the behavioral tests with significance (*p* < 0.05). *p < 0.05 vs. sham, #*p* < 0.05 vs. vehicle, *n* = 8 per group in panels **(A–C)**. Scale bar = 100 μm; *n* = 6 per group in panel **(D)**; *n* = 10 per group in panels **(E–G)**.

As demonstrated in Figures 6A,B, the Y27632 induced less extravasated dye in injured hemisphere at both 24 and 72 h post-CCI (*p* < 0.05) and attenuated post-traumatic brain water content with significance (*p* < 0.05). For inflammatory response, neutrophil number surrounding the lesion area was significantly decreased by Y27632 treatment (*p* < 0.05) (Figure 6C). Furthermore, Y27632 significantly reduced protein levels of IL-1β and TNF-α as compared to the vehicle group (*p* < 0.05) (Figure 6D). Finally, the functional results showed Y27632 induced the overall improvements in all the behavioral tests with significance (*p* < 0.05) (Figures 6E–G).

## Discussion

It is well known that endothelial dysfunction occurs right after brain injury and plays a central role in the secondary response, including BBB disruption, vasogenic edema, and inflammation. Recent studies imply the potential of vascular remodeling by ANXA1 based on the roles of ANXA1 in endothelial function (Ikebuchi and Waisman, 1990; Hubaishy et al., 1995; Lee et al., 2004; Su et al., 2010). Therefore, we hypothesized that exogenous ANXA1 administration may stabilize BBB integrity, decreasing cerebral edema and inflammatory response following TBI. After optimization of dose and timing protocols, the BBB protection by ANXA1 was strongly validated, and an administration protocol of 1 μg/kg at 1 h after operation was selected. Subsequent results demonstrate that ANXA1 treatment improved the neurological outcomes following CCI, correlated with alleviated BBB leakage, cerebral edema, and systemic infiltrating inflammation.

Recent studies indicated the protection of ANXA1 on BBB (McArthur et al., 2016), which is supported by our current results in CCI model. Endogenous ANXA1 response to CCI initiated until 24 h post-injury, whereas rANXA1 treatment at 3 h post-CCI helped restore BBB integrity, indicating an encouraging time window for TBI treatment. Among different time frames, rANXA1 treatment at 1 h post-CCI induced minimal BBB leakage, highlighting the importance of early intervention. In principle, the permeability of BBB is largely based on the function of endothelial junctions (Bose et al., 2018). Thus, the benefits on BBB here may be derived from preserved junctional proteins by rANXA1. Following TBI, BBB disruption permits movement of water from vasculature to the extracellular space in response to elevated osmotic gradient generated by the leakage of vascular components into the brain parenchyma, leading to vasogenic edema (Donkin and Vink, 2010; Luh et al., 2019). As a direct consequence of BBB leakage, edema is responsible for 50% of patient deaths decrease following TBI (Feickert et al., 1999; Marmarou., 2003). In the present study, rANXA1 treatment induced decreased edema in ipsilateral hemispheres following CCI, consistent with the attenuated neurological impairments.

In addition to endothelial cytoskeleton, systemic infiltrating inflammation was also believed to account for BBB disruption. For example, intracerebral administration of IL-1β can induce BBB leakage and related vasogenic edema (Holmin and Mathiesen, 2000; Wang et al., 2020). As a hallmark of systemic infiltrating inflammation following TBI, neutrophil recruited to the inflammatory site requires adhesion to endothelial cells and then cross the blood vessel wall (Sugimoto et al., 2016), which is orchestrated by various molecules expressed by endothelial cells (Subramanian et al., 2016). For example, CD99 has been most extensively studied for its role in neutrophil transmigration, whereas other molecules, notably ICAM-1, VCAM-1, and E-selectin, were known to potentiate neutrophil adhesion (Muller, 2013). Here we showed suppressed neutrophil infiltration and inflammatory cytokines by rANXA1 treatment, partially due to the decreased adhesive and transmigrating capabilities within endothelium. In addition, a previous study also proved that ANXA1 is associated with microglia activity (Liu et al., 2016); thus, ANXA1 may influence inflammatory cytokine expression by acting directly on microglia, as well as reducing neutrophil infiltration.

As a member of Rho family of GTPases, activated RhoA is believed to increase BBB permeability through the destabilization of endothelial actin proteins and tight junction proteins (Terry et al., 2010). However, conflicting results from recent studies seemed to challenge our understanding on it, that local RhoA activation initiates the formation of contractile F-actin structures surrounding neutrophil-induced endothelial pores, preventing plasma leakage during neutrophil diapedesis (Heemskerk et al., 2016). Here we showed active RhoA was suppressed by rANXA1 treatment following CCI, consistent with decreased BBB leakage and junctional proteins loss by rANXA1, suggesting that the junctional restoration from RhoA inhibition outweighs its effect on endothelial pore formation. Furthermore, RhoA/ROCK pathway was lastly reported to affect adhesive molecules expression, and RhoA blockade ameliorated macrophage transfer to the endothelium (Huang et al., 2012). Our studies using isolated microvessels from injured hemisphere showed that RhoA acted to promote neutrophil-endothelial interaction through VCAM-1, ICAM-1, and CD99, which was negatively regulated by ANXA1.

**FIGURE 7:**
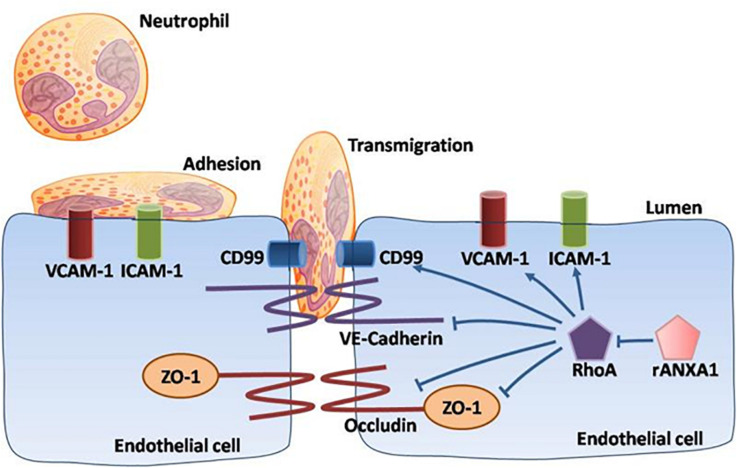
Schematic of ANXA1 modulation of endothelial remodeling. A multipotent role of rANXA1 in microvascular modulation (junctional barrier preservation, endothelial–neutrophil communicating inhibition) in the acute phase following CCI, efficiently correcting BBB disruption and inflammatory cascades.

Overall, our data demonstrated a multipotent role of rANXA1 in microvascular modulation (junctional barrier preservation, endothelial–neutrophil communicating inhibition) in the acute phase following CCI, efficiently correcting BBB disruption and inflammatory cascades (Figure 7). Moreover, rANXA1 treatment exhibited beneficial effect on neurological outcomes, but not on lesion cavity volume. It is acceptable because lesion size might be more associated with primary brain injury following trauma, so it is difficult to be changed by secondary medical intervention. The functional improvements by rANXA1 can be equally achieved by RhoA inhibition. Taken together, these findings suggest that exogenous rANXA1 inhibits BBB disruption and systemic infiltrating inflammation through RhoA inhibition, hence improving functional outcomes in CCI mice. For translational practice, our results provide a novel and practical agent with two advantages in TBI therapy, i.e., ideal administration pathway (intravenous) and effective time window (3 h). It would be worthwhile to seek more potentials of ANXA1 in chronic endothelial events. In addition, cerebral hemorrhage was also considered to be closely associated with the integrity of the BBB. For example, matrix metalloproteinases have been shown to directly disrupt tight junctions, which dramatically altered water reflectance and solute permeability of the BBB, exacerbating brain edema and hemorrhagic contusion expansion (Terry et al., 2010). As the BBB protection by ANXA1 was strongly validated by our study, ANXA1 may play a role on hemorrhagic contusion expansion.

## Conclusion

Taken together, these findings suggest that exogenous rANXA1 inhibits BBB disruption and systemic infiltrating inflammation through RhoA inhibition, hence improving functional outcomes in CCI mice. For translational practice, our results provide a novel and practical agent with two advantages, i.e., ideal administration pathway (intravenous) and effective time window (3 h). Therefore, ANXA1 intervention could be an encouraging target in the TBI therapy.

## Data Availability Statement

The original contributions presented in the study are included in the article/Supplementary Material, further inquiries can be directed to the corresponding author/s.

## Ethics Statement

The animal study was reviewed and approved by the Animal Care and Use Committee of Chongqing Medical University. Written informed consent was obtained from the owners for the participation of their animals in this study.

## Author Contributions

CoC drafted the manuscript and acquired funding for the study. HL, YJ, YW, JH, JZ, ZH, and ZW collected the data for this study. CoC and CeC analyzed and interpreted the data. YD edited the language of manuscript. XS formulated the study concept. ZH designed the study and revised the manuscript. All authors contributed to the article and approved the submitted version.

## Conflict of Interest

The authors declare that the research was conducted in the absence of any commercial or financial relationships that could be construed as a potential conflict of interest.

## Supplementary Material

Supplementary Figure 1 The temporal changes of neutrophil infiltration following CCI. Almost no infiltrated neutrophil was seen in the sham group. The number of infiltrated neutrophils surrounding the lesion area increased maximally at day 3 (*p* < 0.05). Representative images and quantitative analysis of neutrophil infiltration surrounding the lesion area at the preinjury day and days 1, 3, and 7 after CCI. ^∗^*p* < 0.05 vs. sham, *n* = 8 per group, scale bar = 100 μm.
