# Supplementary figures and images for: Endothelial Regulation by Exogenous Annexin A1 in Inflammatory Response and BBB Integrity Following Traumatic Brain Injury
Source: Front Neurosci. 2021 Feb 18;15:627110. doi: 10.3389/fnins.2021.627110 (PMC7930239; doi:10.3389/fnins.2021.627110)

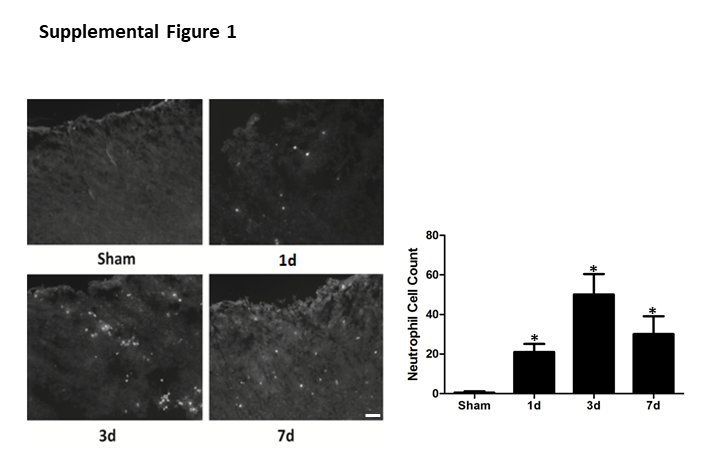

Supplement: Supplementary Figure 1 — The temporal changes of neutrophil infiltration following CCI. Almost no infiltrated neutrophil was seen in the sham group. The number of infiltrated neutrophils surrounding the lesion area increased maximally at day 3 (p < 0.05). Representative images and quantitative analysis of neutrophil infiltration surrounding the lesion area at the preinjury day and days 1, 3, and 7 after CCI. ∗p < 0.05 vs. sham, n = 8 per group, scale bar = 100 μm. [file Image_1.TIF]
